# An epidermal growth factor receptor compound mutation of L858R with S768I in advanced non-small-cell lung cancer: a case report

**DOI:** 10.1186/s13256-024-04422-5

**Published:** 2024-03-18

**Authors:** Sara Boukansa, Ismail Mouhrach, Fatima El Agy, Laila Bouguenouch, Mounia Serraj, Bouchra Amara, Yassine Ouadnouni, Mohamed Smahi, Badreeddine Alami, Nawfel Mellas, Zineb Benbrahim, Hinde El Fatemi

**Affiliations:** 1 Laboratory of Biomedical and Translational Research, Faculty of Medicine and Pharmacy, Sidi Mohamed Ben Abdellah University, Fez, Morocco; 2 Laboratory of Anatomic Pathology and Molecular Pathology, University Hospital Hassan II, Sidi Mohamed Ben Abdellah University, Fez, Morocco; 3 Unit of Medical Genetics and Oncogenetics, University Hospital Hassan II, Sidi Mohamed Ben Abdellah University, Fez, Morocco; 4 Department of Pneumology, University Hospital Hassan II, Sidi Mohamed Ben Abdellah University, Fez, Morocco; 5 Department of Thoracic Surgery, University Hospital Hassan II, Sidi Mohamed Ben Abdellah University, Fez, Morocco; 6 Department of Radiology, University Hospital Hassan II, Sidi Mohamed Ben Abdellah University, Fez, Morocco; 7 Department of Oncology, University Hospital Hassan II, Sidi Mohamed Ben Abdellah University, Fez, Morocco

**Keywords:** *EGFR* mutation, L858R, S768I, Afatinib, Case report

## Abstract

**Background:**

In the current treatment landscape for non-small cell lung cancers, epidermal growth factor receptor-tyrosine kinase inhibitors have emerged as a well-established treatment option for patients with advanced or metastatic disease. This is particularly true for those with commonly occurring epidermal growth factor receptor mutations. However, the therapeutic efficacy of these agents for so-called rare epidermal growth factor receptor mutations, and in particular those characterized by a high degree of complexity, such as double mutations, remains a subject of clinical uncertainty.

**Case presentation:**

In this context, we present the case of a 64-year-old man of Moroccan descent, a lifelong non-smoker, diagnosed with metastatic non-small cell lung cancer characterized by a complex epidermal growth factor receptor mutation encompassing L858R and S768I. The patient subsequently underwent afatinib-based treatment, showing notable clinical results. These included a remarkable overall survival of 51 months, with a median progression-free survival of more than 39 months.

**Conclusions:**

This case report is a compelling testimony to the evolving therapeutic landscape of non-small cell lung cancers, providing valuable insight into the potential therapeutic efficacy of epidermal growth factor receptor-tyrosine kinase inhibitors in the realm of rare and complex epidermal growth factor receptor mutations.

## Introduction

Lung cancer is ranked as the most common cause of cancer-related death worldwide. Non-small cell lung cancers (NSCLCs) are currently the most prevalent form, accounting for approximately 80–85% of all lung cancer cases [1]. The majority of patients diagnosed with lung adenocarcinoma are often in advanced stages (stage IV) and derive modest benefit from nonselective cytotoxic chemotherapy [2]. However, the discovery of epidermal growth factor receptor (*EGFR*) mutations in 2004 has revolutionized the treatment of these patients [3]. Patients with NSCLC with an activating mutation within the epidermal growth factor receptor (*EGFR*) exhibit a dramatic response to EGFR-tyrosine kinase inhibitors (EGFR-TKIs) [4]. 80–90% of activated *EGFR* mutations are normally located in exon 19 deletions and L858R substitution in exon 21 [5]. in addition to these known common mutations, there are other remaining rare mutations whose predictive value is still unclear. Some of these are expressed by the coexistence of two common or rare and common mutations together on different exons in the EGFR gene, which are called “compound mutations.” The clinical response of patients with compound *EGFR* mutations to *EGFR*‑TKIs remains unclear. Several studies indicate that individuals with compound EGFR mutations may show reduced responsiveness to TKI therapies compared with those with single mutations [6–9]. Additionally, different treatment efficacies have been associated with different types of *EGFR* compound mutations [10, 11]. While first-generation *EGFR*-TKIs could be a useful treatment option, recent studies suggest considering afatinib and osimertinib for patients with compound EGFR mutations, depending on their specific cases [12, 13]^.^ These alternative therapies require further investigation to understand their effectiveness and optimal application in the specific context of patients with compound *EGFR* mutations.

We present a case report of a patient with common L858R (exon 21) and rare S768I (exon 20) mutations treated with afatinib.

## Case report

**Fig. 1:**
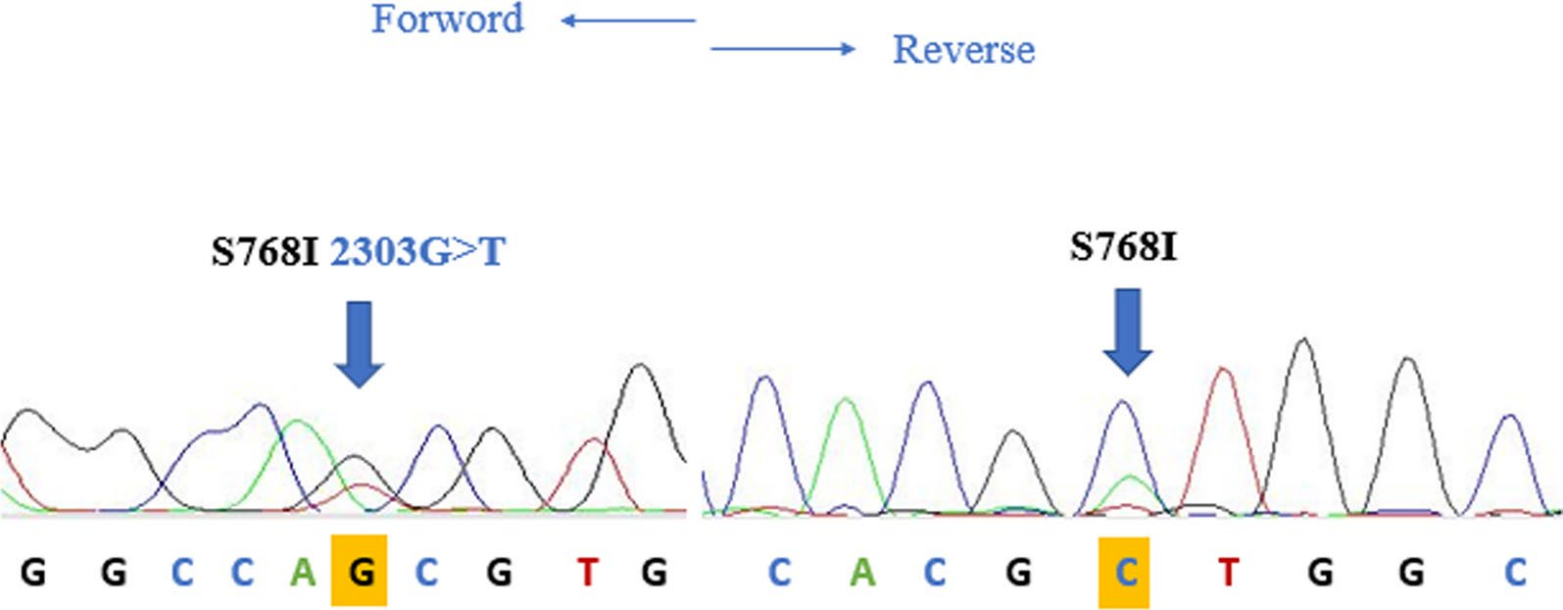
Electropherogram traces of exon 20 S768I positive mutation confirmed by Sanger sequencing

**Fig. 2:**
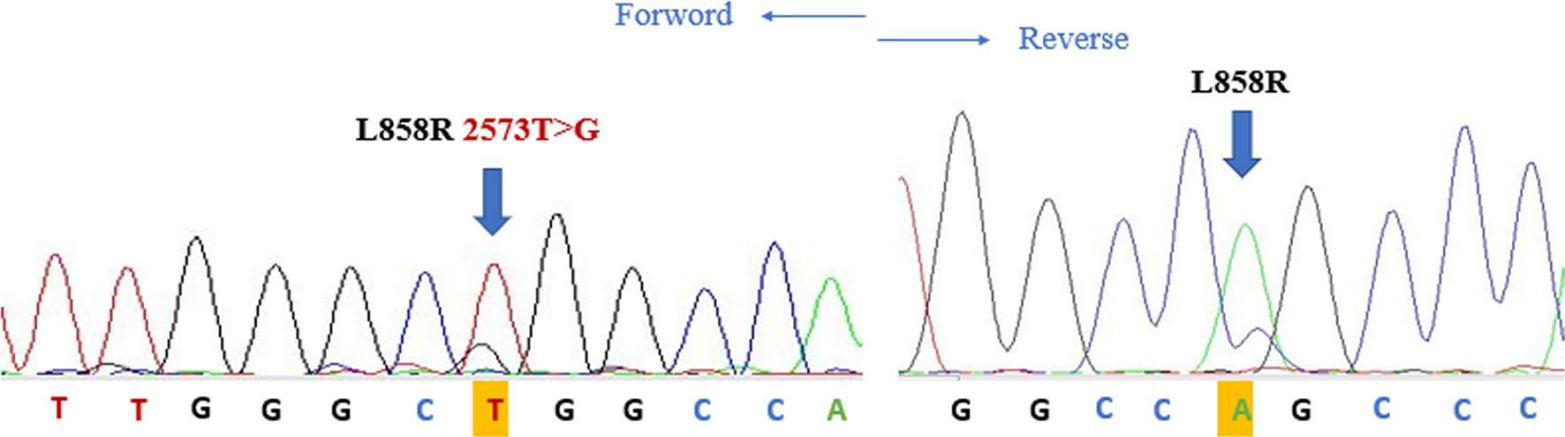
Electropherogram traces of exon 21 L858R positive mutation confirmed by Sanger sequencing

**Fig. 3:**
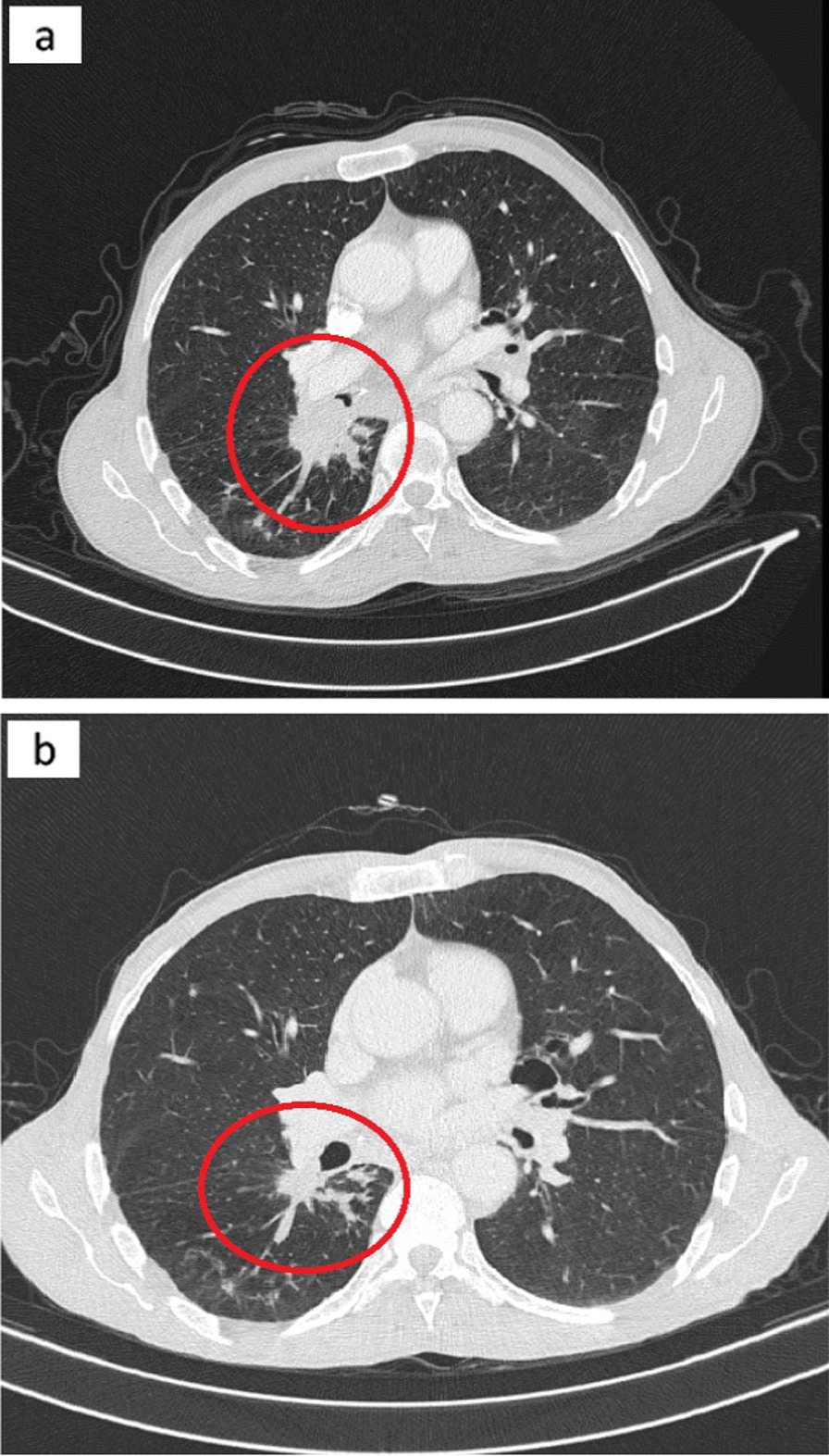
CT scan results showing the primary tumor in the right lower lobe of the lung before treatment (**a**), and the dramatic response 3 months post-afatinib treatment (**b**). The red circle represents the primary tumor before and after treatment

In April 2017, a 64-year-old male of Moroccan descent, a lifelong nonsmoker with no significant medical or family history, presented with a persistent productive cough accompanied by scant hemoptysis and grade 2 dyspnea, which had been ongoing for 3 months. These symptoms were part of a broader clinical picture marked by a progressive decline in his general health status. A comprehensive evaluation, including a chest computed tomography (CT) scan, unveiled a primary tumor mass measuring 95 mm × 69 mm × 66 mm in the lower right lobe of the lung. Additionally, the imaging indicated the presence of lymphangitic carcinomatosis and lymph node involvement on both the ipsilateral and contralateral sides of the thorax, classifying the disease as T4N3M0 according to the TNM staging system (stage IIIc). To establish a definitive histological diagnosis, a transbronchial lung biopsy was performed on the affected right lobe. Subsequently, formalin-fixed paraffin-embedded tissue sections, measuring 4 μm in thickness, were subjected to hematoxylin and eosin saffron (HES) staining, revealing the presence of acinar lung adenocarcinoma. Immunohistochemical staining confirmed the diagnosis, as it exhibited positivity for cytokeratin 7 (CK7) and transcription factor-1 protein (TTF1). The initial therapeutic approach involved first-line chemotherapy, combining cisplatin at a dosage of 75 mg/m^2^ with navelbine at 25 mg/m^2^. Early assessment via CT imaging, performed after the treatment period from 23/11/2017 to 22/12/2017, demonstrated a notable regression in tumor size (from 66 mm × 69 mm × 95 mm to 35 mm × 35 mm × 60 mm) following the initial courses of chemotherapy. However, subsequent CT scans after two cycles of treatment indicated the presence of lung and lymph node metastases, prompting a shift toward palliative chemotherapy. Following this transition, genetic profiling was conducted at the Department of Biomolecular CHU Hassan II to guide personalized therapeutic decisions. DNA extraction was performed from the available histological specimen using the QIAamp DNA FFPE Tissue Kit (Invitrogen), following the manufacturer’s protocols. Genetic analysis employed reverse transcription-polymerase chain reaction (RT-PCR) technology, with the quantitative PCR analysis utilizing the therascreen *EGFR* RGQ PCR Kit. This analysis revealed the presence of an activating co-mutant involving the L858R mutation in exon 21 (c.2573T>G) and the S768I mutation in exon 20 (c.2303G>T, p.S768I). These mutations were subsequently confirmed through SANGER sequencing (Figs. 1, 2). In April 2018, the patient was administered oral afatinib at a daily dose of 40 mg. A follow-up CT scan conducted 3 months post-initiation of afatinib treatment demonstrated a dramatic reduction in tumor size (Fig. 3), accompanied by a significant amelioration of symptoms such as dyspnea, cough, and hemoptysis. Remarkably, the patient exhibited excellent tolerance to afatinib therapy, facilitating a consistent improvement and a relatively slow progression rate of the disease. This response led to a period of disease stabilization until the detection of bone metastases in December 2020. Subsequent months witnessed a state of relative stability in the progression of the primary tumor and associated nodules, until the emergence of a brain metastasis in April 2021. At this juncture, deliberations surrounding potential stereotactic radiotherapy were undertaken; however, the patient opted against surgical intervention. Regrettably, as of the latest available update on 15 August 2022, we have received information confirming the patient’s death.

## Discussion

The management of non-small cell lung cancer (NSCLC) has evolved significantly with a focus on identifying driver mutations, such as *EGFR* and *ALK-EML4,* within tumor samples to guide personalized treatment strategies. The epidermal growth factor receptor (EGFR) is a member of the ErbB family of receptor tyrosine kinases (RTK) [14]. In NSCLC, mutations in the intracellular region of *EGFR* have been observed in a range of 43–89% of cases [15]. Among these mutations, more than 90% are accounted for by *EGFR* TKI-sensitizing mutations, primarily involving exon 19 deletions and the exon 21 L858R substitution [16]. Notably, patients with tumors bearing these activating mutations typically exhibit substantial clinical and radiographic responses to *EGFR* tyrosine kinase inhibitors (TKIs) such as gefitinib, erlotinib, and afatinib [16].

In contrast, the S768I exon 20 substitution (serine replacing isoleucine at codon 768) is described as a rare mutation, representing ~ 1% of cases [17]. The rarity of S768I mutations has posed challenges in defining their role as molecular predictors of TKI response. However, recent studies have shed light on the clinical significance of S768I mutations in lung adenocarcinoma, demonstrating favorable responses to TKIs [18, 19].

Even rarer than the S768I substitution are compound mutations, which encompass not only single exon mutations, but also combinations of double mutations occurring across different exons of the *EGFR* gene. These compound mutations often involve a mix of common and rare mutations (Table 1). In our specific case, the compound mutation was identified as a co-occurrence of L858R exon 21 substitution and the exon 20 S768I mutation. This observation may be attributed to the genetic heterogeneity within EGFR mutation-positive non-small-cell lung cancer, leading to clonal evolution and the emergence of diverse molecular characteristics at the cellular level [20].

**Table 1:** Clinical case summaries—insights from the literature

Case no.	Age, years	Sex	Mutation type	Treatment	Response
Our case	64	Male	L858R + S768I	Afatinib	GR
Svaton *et al*. [31]	63	Female	G719X + S768I	Gefitinib	GR
Masahiro *et al*. [32]	72	Female	G719X + S768I	Afatinib	GR
Yan *et al*. [33]	62	Female	del 2239_2248 + insC + L858R	Erlotinib	PD
Toshiki *et al*. [34]	72	Male	G719X + S768I	Osimertinib	GR
Simionato *et al*. [35]	69	Female	G719A + V769M	Osimertinib	GR
Juan *et al*. [36]	43	Female	19-Del + T790M	Chemotherapy	PD
He *et al*. [37]	64	Female	G724S + R776H	Afatinib	GR
Bao *et al*. [38]	36	Male	R670W + H835L + L833V	Afatinib + osimertinib	GR
Yang *et al*. [39]	N/A	Male	H773L + V774M	Osimertinib	GR
Li *et al*. [40]	53	Female	L833V + H835L	Aumolertinib	GR

*N/A* not available, *GR* good response, *PD* progressive disease

Recent studies have highlighted the complexity and heterogeneity of *EGFR *mutations in NSCLC tumors [20], raising questions about their implications for treatment efficacy. The frequency and response patterns of patients harboring complex mutations remain incompletely defined, primarily due to the limited number of different complex mutations. Generally, compound mutations involving frequent mutations demonstrate similar or improved sensitivity to *EGFR*-TKI therapy [9], and complex mutations, which combine frequent and sparse mutations (non-resistant), tend to exhibit comparable clinical responses to those associated with frequent mutations alone [21].

The L858R substitution emerges as the most prevalent mutation found in conjunction with other rare mutations [21, 22]. Several studies have emphasized the importance of specific compound mutations. For instance, patients with concomitant L858R and V834L or G873E mutations have demonstrated sensitivity to *EGFR*-TKIs comparable to those with single L858R mutations [22]. Additionally, the concomitant del-19 + L858R mutation has been associated with a more favorable clinical response to *EGFR*-TKIs [5].

However, the presence of compound mutations, such as L858R and S768I, in patients with NSCLC presents distinct clinical challenges. Multiple studies have consistently shown that patients with compound *EGFR* mutations tend to exhibit reduced sensitivity to TKI therapies compared with those with single EGFR mutations [6, 7, 23, 24]. Furthermore, emerging evidence suggests that the specific types of compound *EGFR* mutations may be linked to different treatment response patterns [11].

Moreover, the S768I mutation has been associated with resistance to first-generation *EGFR*-TKIs, such as gefitinib and erlotinib, which are commonly used for EGFR-mutated NSCLC [25–27]. In response to the intrinsic resistance displayed by the S768I mutation toward first-generation *EGFR*-TKIs, extensive efforts have been dedicated to exploring alternative therapeutic approaches. Second- and third-generation TKIs, including afatinib and osimertinib, have emerged as promising options, exhibiting notable efficacy against the S768I mutation [25, 28–30]. These agents demonstrate a selective binding affinity for the mutant *EGFR*, effectively surmounting the resistance mechanisms associated with the S768I alteration, thus providing a viable treatment avenue for patients harboring this specific co-mutation.

In the present report, we investigate the efficacy of afatinib *EGFR*-TKI in the context of a 64-year-old Moroccan male patient who was a lifelong nonsmoker and presented with concomitant L858R and S768I mutations. Remarkably, this patient exhibited an overall survival (OS) of 51 months and a median progression-free survival (PFS) exceeding 39 months following afatinib therapy. These outcomes significantly surpass those reported by Ling *et al*. [21], who observed a PFS of 6 months and an OS of 6.5 months in a 70-year-old male smoker (20 pack-years) with L858R + S768I complex mutation treated with gefitinib. This intriguing disparity suggests that the S768I + L858R co-mutation may enhance the interaction between *EGFR* and afatinib compared with gefitinib, resulting in a remarkable clinical response to afatinib TKIs.

## Conclusion

*EGFR* mutation heterogeneity encompasses compound mutations, constituting a distinctive subgroup of patients with NSCLC with varying responses to *EGFR* tyrosine kinase inhibitors. The present report delineates a rare case of acinar lung adenocarcinoma harboring an L858R point mutation in exon 21 and a compound S768I EGFR substitution mutation. Following treatment with afatinib, this patient demonstrated a highly favorable therapeutic response exceeding 38 months. These findings underscore the complexity of EGFR mutations in NSCLC and emphasize the need for tailored treatment strategies to address the diversity of mutational profiles encountered in clinical practice.

## Data Availability

Not applicable.

## References

[1] D’Addario G, Früh M, Reck M, Baumann P, Klepetko W, Felip E (2010). Metastatic non-small-cell lung cancer: ESMO clinical practice guidelines for diagnosis, treatment, and followup. Ann Oncol.

[2] Sandler A, Gray R, Perry MC (2006). Paclitaxel-carboplatin alone or with bevacizumab for non-small-cell lung cancer. N Engl J Med.

[3] Lynch TJ, Bell DW, Sordella R, et al. Activating mutations in the epidermal growth factor receptor underlying responsiveness of non-small-cell lung cancer to gefitinib. N Engl J Med. 2004;350(21):2129-2139. doi:10.1056/NEJMOA040938 10.1056/NEJMoa040938 15118073

[4] Witta SE, Jotte RM, Konduri K (2012). Randomized phase II trial of erlotinib with and without entinostat in patients with advanced non-small-cell lung cancer who progressed on prior chemotherapy. J Clin Oncol.

[5] Keam B, Kim DW, Park JH (2014). Rare and complex mutations of epidermal growth factor receptor, and efficacy of tyrosine kinase inhibitor in patients with non-small cell lung cancer. Int J Clin Oncol.

[6] Kauffmann-Guerrero D, Huber RM, Reu S (2018). NSCLC patients harbouring rare or complex EGFR mutations are more often smokers and might not benefit from first-line tyrosine kinase inhibitor therapy. Respiration.

[7] Tam IYS, Leung ELH, Tin VPC (2009). Double EGFR mutants containing rare EGFR mutant types show reduced in vitro response to gefitinib compared with common activating missense mutations. Mol Cancer Ther.

[8] Kobayashi S, Canepa HM, Bailey AS (2013). Compound EGFR mutations and response to EGFR tyrosine kinase inhibitors. J Thorac Oncol.

[9] Young Kim E, Na Cho E, Surng Park H (2016). Compound EGFR mutation is frequently detected with co-mutations of actionable genes and associated with poor clinical outcome in lung adenocarcinoma. Cancer Biol Ther..

[10] Hata A, Yoshioka H, Fujita S (2010). Complex mutations in the epidermal growth factor receptor gene in non-small cell lung cancer. J Thorac Oncol.

[11] Zhao W, Song A, Xu Y (2023). Rare mutation-dominant compound EGFR-positive NSCLC is associated with enriched kinase domain-resided variants of uncertain significance and poor clinical outcomes. BMC Med.

[12] Kitadai R, Okuma Y (2022). Treatment strategies for non-small cell lung cancer harboring common and uncommon EGFR mutations: drug sensitivity based on exon classification, and structure-function analysis. Cancers (Basel)..

[13] Wu SG, Yu CJ, Yang JCH, Shih JY (2020). The effectiveness of afatinib in patients with lung adenocarcinoma harboring complex epidermal growth factor receptor mutation. Ther Adv Med Oncol..

[14] Bethune G, Bethune D, Ridgway N, Xu Z (2010). Epidermal growth factor receptor (EGFR) in lung cancer: an overview and update. J Thorac Dis..

[15] Gupta R, Dastane AM, Forozan F (2009). Evaluation of EGFR abnormalities in patients with pulmonary adenocarcinoma: the need to test neoplasms with more than one method. Mod Pathol.

[16] Yasuda H, Park E, Yun CH (2013). Structural, biochemical, and clinical characterization of epidermal growth factor receptor (EGFR) exon 20 insertion mutations in lung cancer. Sci Transl Med..

[17] Leventakos K, Kipp BR, Rumilla KM, Winters JL, Yi ES, Mansfield AS (2016). Brief report: S768I mutation in EGFR in patients with lungcancer. J Thorac Oncol.

[18] Yang JCH, Sequist LV, Geater SL (2015). Clinical activity of afatinib in patients with advanced non-small-cell lung cancer harbouring uncommon EGFR mutations: a combined post-hoc analysis of LUX-Lung 2, LUX-Lung 3, and LUX-Lung 6. Lancet Oncol.

[19] Zhu X, Bai Q, Lu Y (2017). Response to tyrosine kinase inhibitors in lung adenocarcinoma with the rare epidermal growth factor receptor mutation S768I: a retrospective analysis and literature review. Target Oncol.

[20] Kohsaka S, Petronczki M, Solca F, Maemondo M (2019). Tumor clonality and resistance mechanisms in EGFR mutation-positive non-small-cell lung cancer: Implications for therapeutic sequencing. Futur Oncol.

[21] Peng L, Song Z, Jiao S (2015). Comparison of uncommon EGFR exon 21 L858R compound mutations with single mutation. Onco Targets Ther.

[22] Li M, Zhou CZ, Yang JJ (2019). The in cis compound EGFR mutations in Chinese advanced non-small cell lung cancer patients. Cancer Biol Ther.

[23] Kobayashi S, Canepa HM, Bailey AS (2013). Compound EGFR mutations and response to EGFR tyrosine kinase inhibitors. J Thorac Oncol.

[24] Kim EY, Cho EN, Park HS (2016). Compound EGFR mutation is frequently detected with co-mutations of actionable genes and associated with poor clinical outcome in lung adenocarcinoma. Cancer Biol Thera..

[25] Xu H, Yang G, Liu R (2022). EGFR uncommon alterations in advanced non-small cell lung cancer and structural insights into sensitivity to diverse tyrosine kinase inhibitors. Front Pharmacol.

[26] Chen K, Yu X, Wang H (2017). Uncommon mutation types of epidermal growth factor receptor and response to EGFR tyrosine kinase inhibitors in Chinese non-small cell lung cancer patients. Cancer Chemother Pharmacol.

[27] Cho JH, Lim SH, An HJ (2020). Osimertinib for patients with non-small-cell lung cancer harboring uncommon EGFR mutations: a multicenter, open-label, phase II trial (KCSG-Lu15-09). J Clin Oncol.

[28] Masuda T, Sunaga N, Kasahara N (2020). Successful afatinib rechallenge in a patient with non-small cell lung cancer harboring EGFR G719C and S768I mutations. Thorac cancer.

[29] Huang CH, Ju JS, Chiu TH (2022). Afatinib treatment in a large real-world cohort of nonsmall cell lung cancer patients with common and uncommon epidermal growth factor receptor mutation. Int J cancer.

[30] Eide IJZ, Stensgaard S, Helland Å (2022). Osimertinib in non-small cell lung cancer with uncommon EGFR-mutations: a post-hoc subgroup analysis with pooled data from two phase II clinical trials. Transl Lung Cancer Res.

[31] Svaton M, Pesek M, Chudacek Z, Vosmiková H (2015). Current two EGFR mutations in lung adenocarcinoma—case report. Klin Onkol.

[32] Watanabe M, Oizumi S, Kiuchi S (2018). The effectiveness of afatinib in a patient with advanced lung adenocarcinoma harboring rare G719X and S768I mutations. Intern Med.

[33] Yang Y, Zhang B, Li R, Liu B, Wang L (2016). EGFR-tyrosine kinase inhibitor treatment in a patient with advanced non-small cell lung cancer and concurrent exon 19 and 21 EGFR mutations: a case report and review of the literature. Oncol Lett.

[34] Morimoto T, Yamasaki K, Shingu T (2023). A rare case of double primary lung adenocarcinomas with uncommon complex EGFR G719X and S768I mutations and pleomorphic carcinoma. Thorac Cancer.

[35] Simionato F, Calvetti L, Cosci M, Scarparo S, Aprile G (2020). Case report: a metabolic complete response to upfront osimertinib in a smoker non-small cell lung cancer patient harbouring EGFR G719A/V769M complex mutation. Oncol Targets Ther.

[36] Falla-Martinez JC, Espinosa D, Baena JC, Rodriguez LX, Sua LF, Zambrano AR (2019). An endothelial growth factor receptor compound mutation of T790M substitution with exon 19 deletion in a previously untreated patient: a case report. J Med Case Rep.

[37] He S-Y, Lin Q-F, Chen J, Yu G-P, Zhang J-L, Shen D (2021). Efficacy of afatinib in a patient with rare EGFR (G724S/R776H) mutations and amplification in lung adenocarcinoma: a case report. World J Clin cases.

[38] Qin BD, Jiao XD, Yuan LY (2018). The effectiveness of afatinib and osimertinib in a Chinese patient with advanced lung adenocarcinoma harboring a rare triple EGFR mutation (R670W/H835L/L833V): a case report and literature review. Oncol Targets Ther.

[39] Yang M, Tong X, Xu X (2018). Case report: osimertinib achieved remarkable and sustained disease control in an advanced non-small-cell lung cancer harboring EGFR H773L/V774M mutation complex. Lung Cancer.

[40] Li L, Huang S, Qin L, Yan N, Shen S, Li X (2023). Successful treatment of lung adenocarcinoma complicated with a rare compound EGFR mutation L833V/H835L using aumolertinib: a case report and literature review. Front Pharmacol.

